# High workload and its connection to health-related quality of life among in-home care workers in northern Sweden during the Covid-19 pandemic

**DOI:** 10.1186/s12889-026-27512-z

**Published:** 2026-05-04

**Authors:** Fredrik Norström, Anita Pettersson-Strömbäck, Magnus Zingmark, Karin Bölenius

**Affiliations:** 1 Department of Epidemiology and Global Health, Umeå University, Umeå, SE-901 87 Sweden; 2 Department of Psychology, Umeå University, Umeå, Sweden; 3 Department of Community Medicine and Rehabilitation, Umeå University, Umeå, Sweden; 4 Department of Nursing, Umeå University, Umeå, Sweden

**Keywords:** Social support at work, Control at work, Strained organisation, Home care

## Abstract

**Background:**

The main aim of this study is to investigate the connection between a high workload and health-related quality of life among in-home care workers in northern Sweden during the COVID-19 pandemic. We also investigate whether social support and control at work can prevent poor health due to high workload.

**Methods:**

A cross-sectional survey was conducted during the pandemic, with 629 (response rate 33 per cent) of an estimated 1,900 in-home care workers responding. Results were compared with a nearly identical survey conducted prior to the pandemic in which 1,154 (response rate 58 per cent) of an estimated 2000 in-home care workers responded. Psychosocial factors were measured using QPSNordic and health-related quality of life using EuroQol 5 Dimensions (EQ-5D). EQ-5D responses were translated into quality-adjusted life year (QALY) scores. Propensity scores were used with absolute risk differences.

**Results:**

During the pandemic, staff with high workload had a statistically significantly (6.2%) lower QALY score (confidence interval 2.2%–10.3%) compared to staff with a normal workload. This was also the case for the usual activities and the anxiety/depression dimensions of EQ-5D. These risk differences were greater, but not statistically significant, during the pandemic than before. The combination of a normal workload and a high degree of control over one’s work appeared to protect against a low QALY score, while social support at work did not seem to be protective.

**Conclusions:**

High workload is related to poorer health-related quality of life. This is mainly attributable to anxiety/depression. In-home care organisations need to manage workload better to prevent poor health among staff during strained situations such as a pandemic. The results of our study indicate that in-home care organisations should increase their readiness to promote opportunities for staff to maintain a high degree of control over their work, in order to counteract variations in workload that ultimately appear to have a negative impact on HRQoL.

**Supplementary Information:**

The online version contains supplementary material available at 10.1186/s12889-026-27512-z.

## Background

In-home care (IHC) services in Organisation for Economic Co-operation and Development (OECD) countries, including Sweden, are facing a challenging labour shortage [1, 2]. According to the National Board of Health and Welfare, the workforce in elderly care in Sweden will need to increase by 28 per cent from 2026 to 2033 [3]. This is attributable to demographic changes and major restructuring in Swedish IHC services over recent decades. This shift has led to IHC services providing more advanced care to enable older people to remain in their homes for longer. Sweden has an aging population, and more older people will require in-home care for longer. Moreover, a significant percentage of those currently employed in IHC services are due to retire in the next 10 years, further exacerbating the staff shortage. Another change over time is the increasing lack of staff continuity; an IHC recipient can expect to encounter many different IHC workers in their home. One Swedish study showed that the average number of recipients a home-care worker visited over the course of a week increased from 8.6 in 2005 to 11.8 in 2015 [4].

In the work environment within Swedish IHC services, staff have reported increased levels of burnout, increased physical workload, health issues and stress of conscience [5–10]. Research has shown that excessive and prolonged stress in the workplace can undermine health on multiple fronts. By introducing the concept of allostatic load, the biological mechanisms underlying stress-related disease have been elucidated, demonstrating how chronic activation of the stress response leads to cumulative physiological wear and tear that predisposes individuals to a range of health conditions [11]. It has been established that occupational demands—when excessively high, frequent, and lacking sufficient opportunities for recovery—elevate the risk of both physical illnesses (e.g., cardiovascular disease, diabetes, immunological dysfunction) and psychological disorders (e.g., depression, burnout) [12]. The importance of maintaining a balance between work demands and recovery, as well as addressing psychosocial stressors in the work environment, has been reinforced by a meta-analysis showing that chronic job strain and long working hours are associated with a 10–40% increased risk of coronary heart disease and stroke, with consistent effects across gender, age, and socioeconomic groups [13]. Similarly, a systematic review linked job strain, low decision latitude, and workplace bullying to the development of depressive symptoms [14]. Workplace stress has been attributed to approximately 120,000 premature deaths annually in the United States [15]. These findings align with physiological models of stress, such as McEwen’s framework of allostatic load, which explains how repeated or chronic environmental challenges lead to dysregulation of neural, endocrine, and immune systems [16].

In our project *Work Environment and Working Conditions* (WeWorC), we demonstrated that, before the COVID-19 pandemic, a high workload was associated with poorer health-related quality of life (HRQoL) among IHC workers, mainly attributable to increased problems in the anxiety/depression dimension of EuroQol 5 Dimensions (EQ-5D) descriptive system [7]. That study used the Johnson and Hall demand-control-support model [17], an extension of Karasek’s demand-control model [18], to evaluate the possible role of control and social support at work in mitigating the adverse health effects of a heavy workload. The results indicate that good social support may mitigate the adverse health consequences of high workload, while control at work did not appear to affect the relationship between workload and health.

Swedish IHC services provide personal care and perform household chores for home care recipients [19]. The services, which are provided pursuant to the Swedish Social Services Act, are largely financed by taxes, although municipalities are permitted to charge users up to €235 per month (2024 figure) [20]. While municipalities are responsible for the financing, delivery and quality assurance of IHC services, the services may be contracted out to private companies [21]. Most of the IHC workers providing IHC are assistant nurses, who have two to three years of health education at a level corresponding to upper secondary school. The next most common category is nursing assistants, who have more limited healthcare training. There are also staff with no formal training [6, 22].

Based on current knowledge, it is evident that achieving a good work environment for IHC staff depends on several factors. To our knowledge, no previous study has specifically investigated the impact of a high workload on those providing IHC during the pandemic or under similarly strained conditions.

The main aim of this study is therefore to investigate the connection between a high workload and HRQoL among IHC workers in northern Sweden during the COVID-19 pandemic. We also investigate whether social support and control at work can prevent poor health due to high workload, and whether the relationship between workload and HRQoL varies between different groups of IHC workers. In our study, we also evaluate whether the social support at work, the control at work, and HRQoL differ between prior to and during the pandemic. Furthermore, we evaluate whether the relationship between workload and health differs between prior to the pandemic, during which this relationship was reported in a previous publication, and during the pandemic.

## Methods

The present study uses data from two cross-sectional surveys of IHC workers in three regions of northern Sweden (Jämtland/Härjedalen, Västerbotten, and Västernorrland), which were administered as paper-based questionnaires. The first survey was commenced in October 2017, prior to the COVID-19 pandemic, and details and results have already been presented [7]. The second survey, conducted during the pandemic in autumn 2021 and spring 2022, is the focus of this paper. The pre-pandemic study provides support for establishing how changes within IHC organisations during the pandemic affected staff.

### Sample and data collection

All 30 municipalities in the three regions were invited to take part in our study, with 16 agreeing to participate in the pre-pandemic survey and 22 in the survey conducted during the pandemic. Fourteen municipalities participated in both surveys. Each municipality coordinated the participation of its own staff and in some municipalities not all IHC units were involved in the study.

The present study uses a 12-page questionnaire titled *A Study of the In-Home Care Work* Environment, which was sent to a contact person at each participating municipality for distribution to IHC workers. In the current study, the only difference between the questionnaires used prior to and during the pandemic was the response alternatives for the question on tenure. Municipalities were recommended to distribute the questionnaire during workplace meetings. The pre-pandemic questionnaire was mainly distributed and responded to during workplace meetings, but for obvious reasons staff generally needed to find other opportunities to respond to the questionnaire during the pandemic. Participating municipalities provided an estimate of the number of IHC workers invited to respond to each survey. Of an estimated 2,000 IHC workers invited to complete the pre-pandemic questionnaire, 1,154 responded (response rate 58%). The corresponding figures for the survey conducted during the pandemic were 1,900 and 629 (response rate 33%).

The questionnaire consisted of questions from the General Nordic Questionnaire for Psychological and Social Factors at Work (QPSNordic), to measure psychosocial factors, EQ-5D to measure health-related quality of life, and questions on gender, age, health education, marital status and tenure in the occupation. The questionnaire provided information about the purpose of our study and that all data was stored securely and handled with full confidentiality. Participants responded anonymously to the surveys, and the municipalities did not provide a list of who had participated.

### Psychosocial measures

In the present study, we used 11 of 129 items (questions) from QPSNordic [23]. These questions were divided into three scales measuring the psychosocial factors *workload*, *social support at work*, and *control at work*. For *workload* four questions belonging to the subscales *quantitative demands*, and *learning demands* were used. For *control at work* four questions from the subscales *control of decision* and *control of work speed* were used, while for *social support at work* three questions from the subscales *support from superior* and *support from co-workers* were used. These questions had five response options: “Very seldom or never”, “Rather seldom”, “Sometimes”, “Rather often”, and “Very often or always”. Questions are presented in Supplementary Table 1 (Table inherited from an earlier publication within the WeWorC project [7]). Cronbach’s alpha showed good reliability for quantative demands (α = 0.81) and weak internal consistency for learning demands (α = 0.51) for responses from participants during the pandemic.

Respondents were defined as having a high workload if the median of their responses was above “sometimes”, which was the third of the five alternatives; otherwise, they were considered to have a normal workload. In sensitivity analyses, we varied the definition of workload by using the questions’ ordinal responses. In the first alternative, high workload was defined as an average score above 3, and in the second alternative, it was defined as an average score above 2.5. Respondents were defined as having a high level of control at work if the median of their responses was at least “sometimes” (the third of five listed alternatives); otherwise, they were considered to have low control. Respondents were defined as having good social support at work if the median of their responses was at least more than “sometimes” (the third of five listed alternatives); otherwise, they were considered to be lacking social support. The cut-offs for high workload, high control at work and good social support have not been previously defined in the literature to our knowledge. Our choices are based on our interpretation of what constitutes a suitable cutoff.

### Health-related quality of life measures

EQ-5D is a validated instrument to measure HRQoL [24]. It includes both a descriptive system and a visual analogue scale (EQ-VAS). The descriptive system consists of five questions corresponding to the five dimensions of health: mobility, self-care, usual activities, pain/discomfort and anxiety/depression. Each dimension is rated on three levels of severity ranging from no problems to extreme problems. Responses to the descriptive system was translated to QALY scores (referred to as EQ-5D index values by the EuroQol Group) using the UK tariff [25]. A QALY score of 0 corresponds to death and a score of 1 to full health.

### Covariates

For the gender variable, woman was used as exposure and compared with man. The few respondents (*n* = 3) with another gender identity were excluded from analyses involving gender. For health education, the outcomes “assistant nurse” and “other health education” were defined as “assistant nurse”, and “nursing assistant” and “no health education” as “other education” and used as exposure. Marital status was classified as either single, used as exposure, or with a partner, where single consisted of all those who indicated “single”, “single with children”, and “with others”. The two surveys use different scales for tenure: the pre-pandemic survey uses ‘less than one year’, ‘1 to 5 years’, ‘6 to 15 years’ and ‘more than 15 years’, whereas in the survey conducted during the pandemic the corresponding alternatives were replaced by ‘after pandemic started’, and ‘1½ to 5 years’. Based on age (calculated from their specified birth year) and tenure, a composite variable was derived whereby at least five years working in IHC (regardless of age) was the exposure. Those who had worked in IHC for over five years were divided into three groups: ‘35 years of age or under’, ‘36–54 years of age’ and ‘55 years of age or over’. Respondents specified their working hours in the survey. In sensitivity analyses, we excluded participants who worked at most 30 hours and at most 35 hours per week, respectively.

### Treatment of missing values in analyses

While the main health outcome used in our analyses was QALY, we also present results for EQ-5D and EQ-VAS. In all analyses, we required valid responses to the outcome (health), the main exposure (workload) and covariates (gender, health education, marital status, and the composite variable of tenure in occupation and age). For analyses of responses during the pandemic, we therefore included 548 (87%) of 629 respondents for analyses of the descriptive system, including QALY, and 551 (88%) of 629 respondents for EQ-VAS analyses. The number of 6 or non-valid responses for the respective variables were: 44 ineligible responses to at least one EQ-5D dimension, 40 for EQ-VAS, 33 ineligible responses to at least one of the workload questions, 8 for gender, 7 for education, 4 for marital status, 4 for tenure and 10 for birth year (used to derive age). Requiring responses for covariates reduced the sample size by 15 participants for analyses of both EQ-5D and EQ-VAS (only 2.7% of participants with valid responses to EQ-5D and workload questions). For the sub-analyses in which social support or control at work were used, an additional 6 participants were excluded from the control at work and 8 from social support at work analyses. Missing value analyses were not conducted due to the relatively low rate of non-responders. A description of participation in both surveys for analyses using EQ-5D is presented in Supplementary Fig. 1.

### Analyses of EQ-5Ds descriptive system

For analyses of the descriptive questions for EQ-5D, responses were dichotomized to “no problems”, and “problems” where the latter alternative included responses corresponding to moderate and severe problems. Analyses were not performed for the EQ-5D dimension self-care, as such a limitation is unlikely in the occupation. This assumption was backed up by questionnaire responses, as only 10 participants stated that they had moderate or severe problems with self-care during the pandemic. Analyses of the relationship between workload and HRQoL during the pandemic were also performed at group level for covariates, if there were at least 100 individuals in the group. Analysis of control at work and social support at work were also performed at group level to evaluate the demand-control-support model.

### Propensity score methodology

The relationship between workload and HRQoL during the pandemic was analysed using propensity scores. These scores estimate the conditional probability of a respondent having a high workload based on covariates. The propensity scores were calculated using logistic regression. Based on the propensity scores, a risk difference was calculated using counterfactual arguments, where the risk difference corresponds to the change in HRQoL when the individual moves from a normal to high workload. A risk difference estimator using inverse probability weighting as suggested by Lunceford and Davidian was used [26]:

$$\begin{aligned}R{D_{IPW}}=&{(\sum\limits_{{i=1}}^{n} {\frac{{{X_i}}}{{P{S_i}}}} )^{ - 1}}\sum\limits_{{i=1}}^{n} {\frac{{{Y_i}{X_i}}}{{P{S_i}}}}\\& - {(\sum\limits_{{i=1}}^{n} {\frac{{1 - {X_i}}}{{1 - P{S_i}}}} )^{ - 1}}\sum\limits_{{i=1}}^{n} {_{i}\frac{{Y(1 - {X_i})}}{{1 - P{S_i}}}} \end{aligned}$$

where *Y* refers to the outcome (HRQoL), *X* to the exposure (workload), *PS* to the propensity score, and *RD* to the risk difference. Using propensity scores in our study results in a quasi-experimental approach. The standardised difference was calculated, both with and without weighting to assess the balance of covariates between the groups with a high and normal workload for each covariate, both before and after propensity scores were applied to them [27]. A standardised difference above 10 per cent is considered by some experts to be a non-negligible imbalance. More details about the methodological approach used are available in Norström et al. [28].

### Statistics

Descriptive statistics were used to present the characteristics of the sample. Pearson’s c^2^ test was used to (i) test whether the exposure variable (workload) was associated with covariates, and (ii) evaluate associations between the study year, i.e. prior to and during the pandemic and other variables. Student’s t-test was used to test differences in age based on workload. Analysis of variance (ANOVA), using the AOV function in R, was used to evaluate whether there was a difference in QALY and EQ-VAS between before and during the pandemic, and whether this could be explained by an interaction between year and workload. This was the only analyses where interactions between explanatory variables were considered. Due to small sample sizes, ANOVA was not used for analyses on a group level. We did not experience problems due to collinearity between variables. Therefore, all candidate variables were kept in the analyses. Stratified analyses were conducted for covariates and psychosocial measures during the pandemic for the relation between workload and the health-related quality of life measures. R Studio was used for statistical analyses (R Studio, Boston, MA), with its GLM procedure used for logistic regression, where confidence intervals were derived with the profile likelihood [29]. Bootstrapping with replacement was used to derive confidence intervals, which corresponded to the 2.5th and 97.5th percentiles, and p-values from 10,000 replicates [30]. Statistical significance was defined at the 5 per cent level.

## Results

### Characteristics of the study populations

During the pandemic, 83 per cent (*n* = 515) were women and 68 per cent (*n* = 425) were married or had a partner (Table 1). It was statistically significantly more common for those with a low level of control at work to have a high workload (*n* = 132; 38%) than those with a high level of control (*n* = 53; 21%). It was also statistically significantly more common for those with a low level of social support (*n* = 93; 39%) to have a high workload compared with those with a high level of social support (*n* = 93; 26%). Those with a high workload also had a statistically significantly lower mean age (44 years) than those with a normal workload (45 years).

**Table 1:** Characteristics of the study population during the pandemic divided into workload (*n* = 629)

	High workload (*n* = 186)			Normal workload (*n* = 410)			Unknown workload (*n* = 33)			*p*
	*n*		%	*n*		%	*n*		%	
Gender										
* Man (n = 106)*	26		25%	74		70%	6		5.7%	0.210
* Woman (n = 515)*	160		31%	329		64%	26		5.0%	
Marital status										
* Married (n = 425)*	126		30%	283		67%	16		3.8%	0.692
* Single (n = 200)*	60		30%	125		62%	15		7.5%	
Tenure										
* After pandemic started (n = 40)*	8		20%	31		78%	1		2.5%	0.442
* 1½*–*5 years (n = 183)*	58		32%	113		62%	12		6.6%	
* 6*–*15 years (n = 186)*	56		30%	122		66%	8		4.3%	
* > 15 years (n = 216)*	63		29%	142		66%	11		5.1%	
Health education										
* Assistant nurse (n = 456)*	131		29%	303		66%	22		4.8%	0.381
* Other education (n = 166)*	53		32%	103		62%	10		6.0%	
Employment form										
* Permanent (n = 572)*	164		29%	377		66%	31		5.4%	0.216
* Temporary (n = 32)*	14		44%	17		53%	1		3.1%	
* By the hour (n = 24)*	8		33%	16		67%	0			
Tenure and age										
* Up to five years of experience (n = 221)*	64		29%	144		65%	13		5.9%	0.995
* More than five years of experience & ≤ 35 years of age (n = 58)*	17		29%	39		67%	2		3.4%	
* More than five years of experience & 36–54 years of age (n = 182)*	56		31%	120		66%	6		3.3%	
* More than five years of experience & ≥ 55 years of age (n = 157)*	46		29%	100		64%	11		7.0%	
Control										
* Low (n = 352)*	132		38%	212		60%	8		2.2%	< 0.001
* High (n = 253)*	53		21%	191		75%	9		3.6%	
Social support										
* Low (n = 241)*	93		39%	142		59%	6		2.5%	0.001
*High (n = 364)*	93		26%	259		71%	12		3.3%	
	*mean*	median	SD	mean	median	SD	mean	median	SD	
*Age (n = 184/403/32)*	*44*	45	13	45	48	13	44	47	14	0.001

*SD* Standard deviation

Missing data is only presented for the workload variable. Three participants responded “other gender” and they are not presented in the table due to the low count. Tests are conducted comparing high and normal workload

As presented in Table 1 in a previous publication from the WeWorC project [31], statistically significantly more participants were foreign-born (8.9 per cent compared with 6.0 per cent) and had permanent positions (91 per cent compared with 84 per cent) during the pandemic than in the pre-pandemic survey. It was also reported that statistically significantly more of the working time was spent in teams (median 20% compared with a median of 16%) and the median age was higher (47 years compared with 45 years) among participants in the pandemic than in the pre-pandemic survey.

### Workload

During the pandemic, the main challenges related to workload were the uneven distribution of work, experienced by 23 per cent (n = 141) of respondents at least “rather often”, and having too much to do, experienced by 28 per cent (n = 170) of respondents at least “rather often” (Table 2). Few respondents mentioned difficult work tasks (n = 10) or work tasks (n = 34) for which they were inadequately trained. According to our definition of high workload, 31 per cent (n = 186) were classified as having a high workload, representing a slight, non-significant increase (*p* = 0.179) compared with 28 per cent prior to the pandemic. For none of the questions covering workload was there a statistically significant association between the question and survey year.

**Table 2:** Workload, control at work and social support at work prior to (n = 1154) and during the pandemic (n = 629)

	Prior to pandemic (2017)									During pandemic (2021)										*p*
	Seldom			Sometimes			Often			Seldom		Sometimes						Often		
	n	%		n	%		n		%	n	%	n			%			n	%	
*Workload*																				
* Unevenly distributed workload*	416	37%		491	43%		222		20%	203	33%	264			43%			141	23%	0.159
* Too much to do*	334	29%		523	46%		281		25%	172	28%	271			44%			170	28%	0.381
* Too difficult work tasks*	1024	91%		95	8.4%		12		1.1%	545	88%	61			9.9%			10	1.6%	0.333
* Work tasks requiring more education*	740	65%		314	28%		81		7.1%	430	70%	151			25%			34	5.5%	0.113
*Control at work*																				
* Influence the amount of work*	567	50%		396	35%		169		15%	324	53%	193			32%			94	15%	0.354
* Influence important decisions*	316	28%		531	47%		286		25%	187	31%	265			43%			160	26%	0.331
* Decide work rate*	524	46%		322	28%		299		25%	304	50%	160			26%			148	24%	0.373
* Decide when to take a break*	688	61%		298	26%		148		13%	383	63%	148			24%			81	13%	0.628
*Social support at work*																				
* Support from colleagues*	28	2.5%		160	14%		947		83%	47	7.7%	118			19%			449	73%	< 0.001
* Support from immediate manager*	122	11%		236	21%		774		68%	73	12%	172			28%			364	60%	0.001
* Appreciation from immediate manager*	250	22%		324	29%		558		49%	133	22%	198			32%			279	46%	0.223
*Summary measure*																				
* Workload*	Normal; n (%)					High; n (%)				Normal; n (%)						High; n (%)				
	798 (72%)					312 (28%)				410 (69%)						186 (31%)				0.179
* Control at work*	Low; n (%)					High; n (%)				Low; n (%)						High; n (%)				
	617 (55%)					495 (45%)				352 (58%)						253 (42%)				0.282
* Social support at work*	Low; n (%)					High; n (%)				Low; n (%)						High; n (%)				
	355 (32%)					770 (68%)				241 (40%)						364 (60%)				0.001

Seldom corresponds to the response alternatives: “Very seldom or never” and “Rather seldom”. Often corresponds to the response alternatives: “Rather often” and “Very often or always”. The summary measures are based on questions about workload, control and social support

### Health-related quality of life

At most 10 respondents reported extreme problems in the dimensions mobility, self-care and usual activities of the EQ-5D descriptive system during the pandemic and only 8.6 per cent (n = 50) reported at least some problems in any of these dimensions (Table 3). It was common for respondents to report some problems in the dimensions pain/discomfort (62%; n = 357) and anxiety/depression (37%; n = 215), although few reported extreme problems (26 respondents in the dimension pain/discomfort and 15 in the dimension anxiety/depression).

**Table 3:** Responses to EuroQol 5 dimensions (n = 596)

		High workload (n = 186)					Normal workload (n = 410)					
		n				%	n					%
Mobility												
* No problems (n = 536)*		161				88%	375					93%
* Some problems (n = 49)*		22				12%	27					6.7%
* Extreme problems (n = 0)*		0					0					
Self-care												
* No problems (n = 568)*		176				98%	392					98%
* Some problems (n = 6)*		2				1.1%	4					1.0%
* Extreme problems (n = 4)*		2				1.1%	2					0.5%
Usual activities												
* No problems (n = 532)*		159				88%	373					93%
* Some problems (n = 46)*		19				10%	27					6.7%
* Extreme problems (n = 4)*		3				1.7%	1					0.2%
Pain/discomfort												
* No problems (n = 222)*		61				34%	161					40%
* Some problems (n = 331)*		107				59%	224					56%
* Extreme problems (n = 26)*		12				6.7%	14					3.5%
Anxiety/depression												
* No problems (n = 363)*		86				49%	277					69%
* Some problems (n = 200)*		85				48%	115					29%
* Extreme problems (n = 15)*		6				3.3%	9					2.2%
	mean				median	SD^b^	mean		median			SD^b^
QALY score^a^ (n = 563)	0.75				0.80	0.24	0.81		0.80			0.19
EQ-VAS^c^ (n = 566)	70.9				75	19.4	77.6		80			16.6

^a^Quality-adjusted life year score, 174 with a high workload and 389 with a normal workload had valid responses to the five descriptive questions in the EuroQol 5 dimensions

^b^Standard deviation

^c^EuroQol Visual Analogue Scale (EQ-VAS), for which 178 with a high workload and 388 with a normal workload had valid responses

### Workload and health-related quality of life

During the pandemic, there was a statistically significantly lower QALY score (6.2%, confidence interval − 10.3% to − 2.2%) associated with a high workload compared to a normal workload (Table 4). Those with a high workload also reported statistically significantly poorer health on the EQ-VAS scale (6.9 lower, confidence interval − 10.3 to − 3.7) compared to those with a normal workload. For EQ-5D, statistically significantly poorer HRQoL was reported by those with a high workload than those with a normal workload in the dimensions usual activities (5.5% more with problems, confidence interval 0.0% to 11%, *p* < 0.05) and anxiety/depression (21% more with problems, confidence interval 12% to 30%). There were no statistically significant differences in HRQoL outcomes associated with workload in the dimensions mobility and pain/discomfort.

**Table 4:** Impact of a high workload on the health-related quality of life of in-home care workers during the pandemic (n = 548)^a^

*Health measure*	Risk difference^b^	Confidence interval		*p*	
*Quality-adjusted life years (QALY) score*	−0.062^c^	[-0.103; -0.022]		0.003	
*EQ-5D*^*d*^ *- Mobility*	0.048^e^	[ -0.004; 0.104]		0.075	
*EQ-5D*^*d*^ *- Usual activities*	0.055^e^	[0.000; 0.114]		0.050^f^	
*EQ-5D*^*d*^ *- Pain/discomfort*	0.058^e^	[-0.030; 0.148]		0.200	
*EQ-5D*^*d*^ *- Anxiety/depression*	0.210^e^	[0.120; 0.298]		< 0.001	
*EQ-VAS* ^*g*^	−6.94^c^	[-10.2; -3.67]		< 0.001	

^a^Of whom 171 are defined as having a high workload and 377 a normal workload

^b^Propensity scores were derived from gender, education level, marital status and a variable combining age and tenure in occupation

^c^A risk difference below 0 means that, compared to a normal workload, a high workload has a lower mean QALY score

^d^EuroQol 5 dimensions. Responses are dichotomized to no problems and at least moderate problems. Problems with each of the dimensions were: 43 for mobility, 48 for usual activities, 334 for pain/discomfort and 206 for anxiety/depression

^e^A risk difference above 0 means that, compared to a normal workload, a high workload leads to more problems in terms of health-related quality of life

^f^The p-value was below 0.05 (*p* = 0.049995)

^g^EuroQol Visual Analogue Scale, n = 551 respondents of whom 174 are defined as having a high workload and 377 a normal workload

Summary statistics for EQ-5D (descriptive dimensions, QALY score and EQ-VAS) and study year are presented in Supplementary Table 2. There was no statistically significant interaction between workload and study year for QALY (*p* = 0.27) and EQ-VAS (*p* = 0.62). We are therefore unable to confirm that a high workload had a greater or lesser adverse health effect. However, the mean QALY score was statistically significantly lower during the pandemic than prior to the pandemic (*p* = 0.004), decreasing from 0.80 (standard deviation (SD) 0.19) to 0.75 (SD 0.24) for those with a high workload and from 0.83 (SD 0.17) to 0.81 (SD 0.19) for those with normal workload. The mean EQ-VAS was also statistically significantly lower (*p* < 0.001) during the pandemic, decreasing from 75 (SD 25) to 71 (SD 19) for those with high workload and from 81 (SD 15) to 78 (SD 17) for those with normal workload. During the pandemic, there was a statistically significant increase in reported problems in the dimensions usual activities (*p* = 0.017) and anxiety/depression (*p* < 0.001). In Supplementary Table 3, group-level results are presented for mean QALY scores for high and normal workload, reported separately for the pre-pandemic and pandemic surveys.

The application of propensity scores led to a better balance between the outcomes of the covariates in the workload groups in the analyses conducted. The standardised difference ranged from 0.6 to 12 per cent when weights for the QALY score estimates were not applied and from 0.2 to 0.7 per cent when propensity score weights were applied (Supplementary Table 4). However, for tenure and age, which were used as a composite variable in the derivation of the propensity scores, the balance was not optimal for tenure of at most 1½ years and tenure between 1½ and 5 years, as the standardised difference was over 10 per cent. The balance was also slightly high for the age variable.

### Workload and health-related quality of life at group level during the pandemic

Stratified analyses during the pandemic revealed a statistically significantly poorer QALY among married participants (*p* = 0.015) with a high workload compared with those with normal workload, whereas no statistically significant difference was found among single participants (*p* = 0.056), despite the effect estimate being larger for singles (Table 5). The same comparisons showed statistically significantly poorer EQ-VAS scores for both groups (Table 6), with a larger effect estimate for married participants (*p* = 0.001) than for singles (*p* = 0.043). For both the pain/discomfort and the anxiety/depression dimensions, married and single participants showed the same statistical significances (Table 7). However, the point estimates for the anxiety/depression dimension differed substantially, with 29 per cent more singles reporting problems (*p* < 0.001) and 19 per cent more married reporting problems (*p* < 0.001) when high workload was compared with normal workload. The difference in point estimates was even larger for the non-significant results for the pain/discomfort dimension.

**Table 5:** Stratified results showing the adverse health effects of a high workload on in-home care workers during the pandemic, expressed as quality-adjusted life-year scores (QALY)

Stratification group	Risk difference^a^	Confidence interval	*p*
Gender			
* Man (n = 86)*	n/a		
* Woman (n = 462)*	-0.044	[-0.089; -0.001]	0.045
Marital status			
* Married (n = 383)*	-0.061	[-0.113; -0.018}	0.015
* Single (n = 165)*	-0.064	[-0.138; 0.033]	0.056
Health education			
* Assistant nurse (n = 408)*	-0.063	[-0.112; -0.018]	0.004
* Other education (n = 140)*	-0.052	[-0.138; 0.003]	0.228
Tenure and age			
* Up to five years of experience (n = 187)*	-0.120	[-0.197; -0.048]	< 0.001
* More than five years of experience & ≤ 35 years of age (n = 53)*	n/a		
* More than five years of experience & 36*–*54 years of age (n = 168)*	-0.029	[-0.108; 0.045]	0.466
* More than five years of experience & ≥ 55 years of age (n = 140)*	-0.004	[-0.059;0.051]	0.892
Control			
* Low (n = 322)*	-0.039	[-0.090; 0.012]	0.139
* High (n = 220)*	-0.073	[-0.146; -0.004]	0.038
Social support			
* Low (n = 216)*	-0.060	[-0.126; 0.004]	0.067
* High (n = 324)*	-0.047	[-0.102; 0.006]	0.085

^a^A risk difference below 0 means that, compared to a normal workload, a high workload has a lower mean QALY score

n/a – the group was too small to be able to present meaningful estimates

**Table 6:** Stratified results for the adverse health effects of a high workload during the pandemic on the EuroQol 5D Visual Analogue (EQ-VAS) Scale

Stratification group	Risk difference^a^	Confidence interval	*P*-value
Gender			
* Man (n = 86)*	n/a		
* Woman (n = 462)*	-5.93	[-9.57; -2.31]	< 0.001
Marital status			
* Married (n = 383)*	-7.46	[-11.4; -3.56]	0.001
* Single (n = 165)*	-6.54	[-12.9; -0.13]	0.043
Health education			
* Assistant nurse (n = 408)*	-7.26	[-11.1; -3.49]	< 0.001
* Other education (n = 140)*	-5.20	[-12.5; 2.01]	0.154
Tenure and age			
* Up to five years of experience (n = 187)*	-10.8	[-16.8; -4.86]	< 0.001
* More than five years of experience & ≤ 35 years of age (n = 53)*	n/a		
* More than five years of experience & 36*–*54 years of age (n = 168)*	-6.37	[-12.6; 0.02]	0.054
* More than five years of experience & ≥ 55 years of age (n = 140)*	-4.56	[-10.3; 1.14]	0.114
Control^b^			
* Low (n = 322)*	-4.11	[-8.56; 0.39]	0.070
* High (n = 220)*	-8.48	[-13.7; -4.18]	< 0.001
Social support^c^			
* Low (n = 216)*	-5.25	[-10.3; 0.88]	0.091
* High (n = 324)*	-6.51	[-10.7; -2.47]	< 0.001

^a^The risk difference represents the change in mean EQ-VAS score due to high workload in comparison with normal workload. A risk difference below 0 means that, compared to a normal workload, a high workload has a lower mean EQ-VAS score

n/a – the group was too small to be able to present meaningful estimates

**Table 7:** Stratified results for the adverse health effects of a high workload during the pandemic in the EQ-5D dimensions

Stratification group	Risk difference^a^		*p*
*Pain/discomfort*			
Gender			
* Man (n = 86)*	n/a		n/a
* Woman (n = 462)*	0.024		0.612
Marital status			
* Married (n = 383)*	0.022		0.699
* Single (n = 165)*	0.141		0.071
Health education			
* Assistant nurse (n = 408)*	0.040		0.433
* Other education (n = 140)*	0.121		0.182
Tenure and age			
* Up to five years of experience (n = 187)*	0.161		0.046
* More than five years of experience & ≤ 35 years of age (n = 53)*	n/a		n/a
* More than five years of experience & 36*–*54 years of age (n = 168)*	0.021		0.791
* More than five years of experience & ≥ 55 years of age (n = 140)*	-0.049		0.586
Control^b^			
* Low (n = 322)*	0.012		0.825
* High (n = 220)*	0.057		0.539
Social support^c^			
* Low (n = 216)*	0.082		0.199
* High (n = 324)*	-0.006		0.916
*Anxiety/depression*			
Gender			
* Man (n = 86)*	n/a		n/a
* Woman (n = 462)*	0.171		< 0.001
Marital status			
* Married (n = 383)*	0.186		< 0.001
* Single (n = 165)*	0.287		< 0.001
Health education			
* Assistant nurse (n = 408)*	0.219		< 0.001
* Other education (n = 140)*	0.173		0.057
Tenure and age			
* Up to five years of experience (n = 187)*	0.348		< 0.001
* More than five years of experience & ≤ 35 years of age (n = 53)*	n/a		n/a
* More than five years of experience & 36*–*54 years of age (n = 168)*	0.065		0.413
* More than five years of experience & ≥ 55 years of age (n = 140)*	0.212		0.016
Control^b^			
* Low (n = 322)*	0.190		0.001
* High (n = 220)*	0.205		0.031
Social support^c^			
* Low (n = 216)*	0.197		0.005
* High (n = 324)*	0.164		0.006

^a^The risk difference represents the increase in the percentage of individuals reporting health problems due to a high workload

n/a – the group was too small to be able to present meaningful estimates

Interestingly, the stratified results for tenure and age showed that the adverse effects of a high workload were much greater, and statistically significant, among those recently employed (up to five years), who had a 12 per cent lower mean QALY score (*p* < 0.001) compared with those with a normal workload, with 16 per cent more reporting at least some problems with pain/discomfort (*p* = 0.046), and 35 per cent more reporting at least some problems with anxiety/depression (*p* < 0.001). In contrast, the impact of a high workload was limited in both these dimensions for the groups with at least five years of experience and 36 years or older. The only statistically significant results for these two groups in any of the dimensions of health were for anxiety/depression among IHC workers of 55 years of age or over (*p* = 0.016).

For assistant nurses and for women, the same statistical significances were observed as for the total population, whereas no statistically significant findings were noted among participants with an education other than assistant nurse. No estimates were made for the groups *man* and those with *more than five years of experience and ≤ 35 years of age*, as there were too few respondents in these groups.

### Control and social support at work

Between 13 and 26 per cent of respondents reported at least some problems for the different questions related to control at work. The most common issue was exerting influence over important decisions, with only 31 per cent seldom experienced problems in this regard. During the pandemic, 58 per cent of the participants were classified as having a lack of control at work, which represented a slight, non-significant increase (*p* = 0.282) compared with 55 per cent prior to the pandemic. There were no statistically significant changes to how respondents answered questions on control at work prior to and during the pandemic.

During the pandemic, 60 per cent of the participants were classified as having a good social support at work, representing a statistically significant decrease (*p* = 0.001) compared with 68 per cent prior to the pandemic. This decline was explained by statistically significant reductions in social support from colleagues and from the immediate manager: the proportion reporting support from colleagues decreased from 83 per cent to 73 per cent (*p* < 0.001), and the proportion often experiencing support from their immediate manager decreased from 68 per cent to 60 per cent (*p* = 0.001). Less than half (46%) of the respondents felt appreciated by their immediate manager during the pandemic.

### Health-related quality of life in relation to the job demand-control-support model

Individuals who were able to control their own work to a large degree had a statistically significantly (*p* = 0.038) lower (7.3 per cent) QALY score if they had a high workload compared to having a normal workload (Table 5). The corresponding figure for those with little opportunity to control their own work was a statistically non-significant 3.9 per cent. Those with a normal workload who were able to largely control their work had a higher mean QALY score (0.84) than other groups during the pandemic (Supplementary Table 3). Both the group with high (20.5% difference, *p* = 0.031) and the group with low (19.0% difference, *p* = 0.001) control at work reported a similar, statistically significant increase in anxiety/depression when they had a high rather than normal workload (Table 7).

The groups with low and high social support at work both had a lower QALY score when they had a high rather than normal workload, although this was statistically non-significant (Table 5). While the group with a high level of social support at work (-6.5 difference, *p* < 0.001) had a statistically significantly higher EQ-VAS score, but not the group with low social support at work (Table 6). The difference in estimates between groups based on social support at work was limited. A high workload had a statistically significant adverse effect on both groups in the anxiety/depression dimension (Table 7).

### Sensitivity analyses

In our sensitivity analyses based on the alternative definitions of workload (Supplementary Table 5), the first definition resulted in only 48 participants being classified as having high workload. The absolute risk difference for QALY between high and normal workload was statistically significant and numerically much larger (12%) than in our main analysis (6.2%). For the pain/discomfort dimension, only this definition showed a statistically significant increase in problems between workers with high and normal workload, with the increase being as large as 21 per cent. For the second alternative definition, all effect estimates were larger than in the main analyses, while exhibiting the same statistical significances. Sensitivity analyses based on participants’ working hours showed numerical differences for QALY (ranging from 0.061 to 0.072 lower QALY due to high workload compared to normal workload) and for the anxiety/depression dimension (ranging from 21% more to 26% more with problems due to high workload compared to normal workload), while maintaining the same statistical significances (Supplementary Table 6).

## Discussion

The present study explores connections between workload and HRQoL among IHC workers in northern Sweden during the pandemic. We were able to show that staff with a high workload had lower QALY and EQ-VAS scores than those with a normal workload. Furthermore, IHC workers with a high workload consistently reported more problems in the dimensions usual activities and anxiety/depression during the pandemic. The impact of a high workload compared with a normal workload on HRQoL was also examined prior to the pandemic, yielding the same evidence at the population level. Although effect estimates were larger during the pandemic than before [7], we still found no evidence that a high workload, in relation to a normal workload, led to poorer HRQoL during the pandemic. Our study indicated that maintaining a normal workload and having high control at work may help prevent poorer health. IHC workers experienced a decline in social support in the workplace from 68 per cent pre-pandemic to 60 per cent during the pandemic, while we found no evidence that social support helped buffer the consequences of a high workload.

In the group-level analyses, it was most notable that recently employed IHC workers were affected by a high workload, whereas statistical evidence that a high workload worsened health among other tenure and age groups was limited to the anxiety/depression dimension for experienced workers above 55 years of age. The other notable variation between groups was that there was no evidence of poorer HRQoL due to high workload among those who were not assistant nurses, whereas assistant nurses reported the same problems as for the whole population.

Apart from our previous publications within the WeWorC project [6, 7], we are not aware of any study that has explored HRQoL among IHC workers, either during the pandemic or at other times. A study of care home workers in Spain reported mean QALY and EQ-VAS scores similar to those found among our participants with a high workload (a mean QALY score of 0.77 in the Spanish study compared with 0.75 in ours, and a mean EQ-VAS score of 69.7 compared with our 70.9), implying that care workers elsewhere also faced a challenging situation during the pandemic [32]. Health challenges such as stress and mental health issues have also been reported in other settings, such as nursing homes and residential homes, in both qualitative [33] and quantitative [34] studies conducted during the pandemic. The challenges of workload and stress for staff in residential homes during the pandemic have likewise been reported [35, 36]. However, we are not aware of studies outside our project that have investigated how workload relates to HRQoL or other health measures in IHC services or within other sectors of home care. Hence, we are unable to relate our main findings to other research studies conducted during the COVID-19 pandemic.

Our results showed that a high workload compared with a normal workload, worsened the QALY score by 6.2 per cent, whereas the corresponding difference prior to the pandemic was 3.5 per cent [7]. A QALY difference of 5 per cent means that living in the poorer condition, in our case experiencing a high workload, for 20 years is equal to living 19 years in the better condition, in our case a normal workload. Thus, in health-economic terms, our results from the pandemic can be interpreted as suggesting that it would be worth living one year less over a 20-year period to avoid a high workload. As was the case prior to the pandemic, it was in the usual activities and the anxiety/depression dimensions that IHC workers with a high workload experienced more problems.

To the best of our knowledge, no previous studies have reported results relating HRQoL during the pandemic to the demand-control-support model [17]. A mixed-methods study conducted in Finland [37] and a qualitative study in Norway [38] did, however, produce results demonstrating the importance of IHC workers having control at work during the pandemic. The Finnish study found that both objectively measured and subjectively experienced work demands—such as time pressure, frequent interruptions and role conflicts—were strongly associated with increased stress and reduced job satisfaction among IHC workers [37]. Furthermore, their study concluded that greater autonomy and increased continuity of care – i.e., factors that can be described as perceived control over one’s work – were associated with higher job satisfaction. They related a heightened risk of burnout and turnover of staff to stressors that are exacerbated by a shortage of personnel and an increasing number of care recipients. In the Norwegian study [38], the concept of “organizational anomie” was explored. A disconnect between the ideals of caregiving and the realities imposed by organisational structures was described. Workers reported experiencing feelings of moral distress when, due to time constraints and limited resources, they were unable to provide the level of care they believed was necessary. This moral strain contributed to emotional exhaustion and a diminished sense of professional fulfilment.

Our findings are in line with the Norwegian and Finnish studies [37, 38]. In our study, IHC workers with low levels of control at work appeared to be more adversely affected by a high workload. This conclusion is supported by evidence of a negative impact of 7.3 per cent on the mean QALY score when comparing those with high to those with a normal workload, among individuals with a high level of control at work, whereas the corresponding figure for those with a low level of control at work was 3.9 per cent and not statistically significant. It appears that having both a manageable workload and a high degree of control over one’s work was particularly beneficial for IHC workers during the pandemic – a pattern that was not observed prior to the pandemic. Our results suggest that, within the framework of the demand-control-support model, a high level of control at work may serve as a critical protective resource during periods of heightened occupational stress, whereas social support appears to offer comparatively limited benefit under such conditions. Thus, good social support that seemed to be valuable prior to the pandemic seemed to not be protective during the pandemic [7]. In a Swedish study from 2018 [39], it was demonstrated that high job demands are consistently associated with an increased risk of burnout, irrespective of the availability of workplace support, thus corroborating the importance of reducing job demands and increasing control to support employee well-being within IHC services.

In a research overview of scholarly articles and reports from Swedish government agencies published between 2005 and 2015 [40], the relationships between various organisational and psychosocial factors and work-related as well as health-related outcomes among working women and men were investigated. The authors concluded that organisational and psychosocial factors do influence work- and health-related outcomes, which is in line with how we interpret our results.

### Methodological considerations

The response rate to the survey conducted during the pandemic was low, both in absolute terms and compared with the pre-pandemic survey. There were differences in who responded to the surveys; for instance, the pandemic survey included a higher proportion of IHC workers who were foreign-born, had permanent contracts, and worked night shifts. There is therefore an obvious risk that comparisons between the time periods are biased. However, our main interest lies in presenting the results for the pandemic itself and in them comparing IHC workers with high and normal workload with analyses that adjust for the variables we regard as the most likely to confound this association. The differences in who responded to surveys are therefore of more limited interest in our study. We believe that the differences identified between the two years should reflect actual changes, as they appear logical given the impressions we gained from reports in the media during the pandemic. Comparisons between high and normal workload are likely to be representative for all IHC workers as we would not expect a different relation between non-responders in these groups. However, this possibility cannot be fully ruled out.

These interesting, if somewhat perplexing, deviations may be due to the number of IHC workers in the subgroups being too few or due to confounding effects. Potentially they may also be related to factors such as structural differences in work tasks within IHC services between the groups. Trends were similar in the previous publication from the WeWorC project, albeit much more modest [7]. All group-level analyses, both before and during the pandemic, should be treated with caution as the groups are relatively small, and neither of the studies were dimensioned for these analyses.

Observational studies, and especially cross-sectional studies, have their limitations. To limit confounding effects in our estimates, we used inverse propensity score weighting. The standardised differences for variables after weighting in our main analyses indicated that our approach could handle measured covariates. Still, we cannot rule out that other confounders may affect our estimates. However, as we have included the factors that we consider to be the most likely confounders based on previous research, we expect the bias from not including other variables in the propensity scores to be negligible. Due to the cross-sectional design of the study, we have limited possibilities to understand the causal mechanism. Clearly, it may be the case that IHC managers allocate high workloads based on varying characteristics, such as the age, experience or sex of staff.

We would expect to find that individuals who are ill-equipped to cope with a high workload are on sick leave or have quit the job. Hence those still working in the occupation under a high workload are likely to be those with greater capacity for work. Thus, the high workload may be overrepresented by IHC workers that are better in handling such situation. We would therefore expect those who left the occupation due to a high workload to have a lower QALY score. So, if anything, we would expect our results to underestimate the burden of a high workload. During the pandemic, we encouraged municipalities to invite staff who were on sick leave to participate in the study with only few of them participating. However, we realised that this placed an unreasonable demand on IHC organisations, so we made no additional attempts to reach this group during the pandemic. Due to the expected low rate of IHC workers on sick leave, the prevalence of IHC workers with a high workload is probably underestimated.

As our studies have been conducted in northern Sweden, our results may lack generalisability. That said, our mean QALY scores were similar to previous results produced in Spain among care home workers during the pandemic [32]. Even if it cannot be confirmed, we believe that the results of our study will prove valuable in other settings, both inside and outside Sweden. To the best of our knowledge, these are also the only results available to organisations in similar settings on which to base their ideas about the consequences of high workloads under extraordinary circumstances, as represented by the pandemic.

There is no guideline in the manual to QPSnordic on how workload should be defined [23]. We therefore developed our own definition. To illustrate how the definition of workload may influence the estimates, we conducted sensitivity analyses. Our definition, which requires more pronounced problems to classify a respondent as having a high workload, produced larger observed problems associated with high workload. This suggests that we may underestimate the true burden of a high workload. One may argue that the alternative, more inclusive definition could be preferable. However, we chose our definition to avoid overstating the problem. Moreover, the more inclusive definition would have resulted in a very small sample in the high‑workload group, which would have been an additional limitation. In our main analysis, we chose to include participants with few working hours. A workload may still be challenging even if one does not work full time. Using more restrictive inclusion criteria resulted in higher effect estimates, which again suggests that we may underestimate the true effect of a high workload in our study.

### Policy implications

Swedish IHC services will need to substantially increase their workforces in the near future and also replace staff who are retiring [3]. If they fail to do so, existing staff are likely to face a significant increase in workload. There was clearly nothing IHC services could do to prevent the pandemic. It is reasonable to assume that staff were more highly motivated to go above and beyond the call of duty in such exceptional circumstances than they would be in a similarly strained situation caused solely by a lack of resources. Considering that our study showed that a high workload during the strained situation during the pandemic was associated with poorer HRQoL, it is evident that a similarly high and more permanent increase in workload due to staff shortages would not pose a smaller problem. Therefore, it is important to take action to ensure that sufficient staffing levels can be secured in IHC services. In a report by the Swedish National Board of Health and Welfare, it is noted that many municipalities already fail to recruit sufficient staff, making it urgent to find solutions to prevent IHC workers from experiencing high workloads and the poorer health outcomes associated with them [3]. We also consider it important to invest in improving organisational and psychosocial factors in the workplace for IHC workers, based on results from our own and other studies [40].

## Conclusions

In conclusion, our study reveals a connection between high workload and poorer HRQoL during the pandemic. During the pandemic, it appears that having both a high level of control at work and a normal workload was an important factor in preventing a decline in HRQoL of IHC workers. The results of our study indicate that in-home care organisations should increase their readiness to promote opportunities for staff to maintain a high degree of control over their work, in order to counteract variations in workload that ultimately appear to have a negative impact on HRQoL. An IHC organisation under strain places the health of staff at risk. It is therefore vital that these organisations are well prepared to meet especially challenging working situations such as a pandemic.

## Supplementary Information

Supplementary Material 1.

Supplementary Material 2.

Supplementary Material 3.

## Data Availability

The datasets generated and/or analysed during the current study are not publicly available because the Swedish Data Protection Act (1998:204) does not permit sensitive data on humans to be freely shared. The datasets are available based on ethical permission from the Swedish Ethical Review Authority from one of the authors (Fredrik Norström).

## Abbreviations

ANOVA: Analysis of variance
EQ-5D: EuroQol 5 Dimensions
EQ-VAS: EuroQol visual analogue scale
HRQoL: Health-related quality of life
IHC: In-home care
OECD: Organisation for Economic Co-operation and Development
QALY: Quality-adjusted life-year
QPSNordic: General Nordic Questionnaire for Psychological and Social Factors at Work
WeWorC: Work Environment and Working Conditions
