# Supplementary material for: High workload and its connection to health-related quality of life among in-home care workers in northern Sweden during the Covid-19 pandemic
Source: BMC Public Health. 2026 May 4;26:1468. doi: 10.1186/s12889-026-27512-z (PMC13141269; doi:10.1186/s12889-026-27512-z)
Supplement: Supplementary file 1 — Supplementary Material 1. [file 12889_2026_27512_MOESM1_ESM.docx]

| **Supplementary Table 1**: Scales, subscales and items in a reduced QPSNordic version used in current study. |
| --- |
| **Workload – Quantitative demands**  Is your workload so unevenly distributed that work is getting bogged down?  Do you have too much to do?  **Workload – Learning demands**  Are your work tasks too difficult for you?  Perform tasks for which you would need more education  **Control – Decision**  Can you influence the amount of work you get?  Can you influence decisions that are important to your work?  **Control – Work speed**  Can you decide your work rate yourself?  Can you decide when to take a break?  **Social support – Support from superior**  If you need, do you receive support and assistance with your work from your immediate boss?  Does your immediate boss appreciate your work performance?  **Social support – Support from co-workers**  If you need it, do you receive support and assistance with your work from co-workers? |

Note: all questions had five response alternatives: very seldom or never, rather seldom, sometimes, rather often, and very often or always. Previously published as Table 1 in Sjöberg et al 2020.

**Supplementary Table 2.** Responses to EuroQol 5 dimensions (EQ-5D).

|  | | Before the pandemic (2017) | | | | | During the pandemic (2021-2022) | | | |
| --- | --- | --- | --- | --- | --- | --- | --- | --- | --- | --- |
|  | | n | % | | | | n | | % | |
| **Mobility** | |  | | |  | |  | |  | |
| *No problems* | | 1066 | | 93.6 | | | 559 | | 91.5 | |
| *Some problems* | | 72 | | 6.3 | | | 52 | | 8.5 | |
| *Extreme problems* | | 1 | | 0.1 | | | 0 | | - | |
| **Self-care** | |  | | |  | |  | |  | |
| *No problems* | | 1120 | | 99.4 | | | 592 | | 98.3 | |
| *Some problems* | | 4 | | 0.4 | | | 6 | | 1.0 | |
| *Extreme problems* | | 3 | | 0.3 | | | 4 | | 0.7 | |
| **Usual activities*** | |  | | |  | |  | |  | |
| *No problems* | | 1070 | | 94.4 | | | 558 | | 91.5 | |
| *Some problems* | | 62 | | 5.5 | | | 46 | | 7.5 | |
| *Extreme problems* | | 1 | | 0.1 | | | 6 | | 1.0 | |
| **Pain/discomfort** | |  | | |  | |  | |  | |
| *No problems* | | 460 | | 40.7 | | | 233 | | 38.6 | |
| *Some problems* | | 631 | | 55.9 | | | 344 | | 56.9 | |
| *Extreme problems* | | 39 | | 3.4 | | | 27 | | 4.5 | |
| **Anxiety/depression*** | |  | | |  | |  | |  | |
| *No problems* | | 810 | | 71.7 | | | 385 | | 63.4 | |
| *Some problems* | | 306 | | 27.1 | | | 207 | | 34.1 | |
| *Extreme problems* | | 14 | | 1.2 | | | 15 | | 2.5 | |
|  | |  | |  | | |  | |  | |
|  | mean | | | median | | SD^b^ | mean | median | | SD^b^ |
| **QALY score^a^** | 0.819 | | | 0.796 | | 0.18 | 0.795 | 0.796 | | 0.207 |
| **EQ-VAS^c^** | 79.0 | | | 80 | | 18.8 | 75.7 | 80 | | 17.8 |

^a^ Quality-adjusted life years score. There were 1,110 participants with QALY scores before the pandemic and 585 participants during the pandemic with eligible answers.
^b^ Standard deviation.
^c^ For EuroQol Visual Analogue Scale (EQ-VAS). There were 1,083 participants with QALY scores before the pandemic and 589 participants during the pandemic with eligible answers.
* Statistical significance at the 5% level using Pearson’s χ^2^ test, with “some” and “extreme” problems combined into one group. Due to the small number of respondents reporting problems, no analyses were conducted for “Self-care”.

**Supplementary Table 3.** Quality-adjusted life year (QALY) scores on group-level.

|  | **Before the pandemic** | | | | **During the pandemic** | | | |
| --- | --- | --- | --- | --- | --- | --- | --- | --- |
|  | *High workload* | | *Normal workload* | | *High workload* | | *Normal workload* | |
|  | mean | SD | mean | SD | mean | SD | mean | SD |
| **Gender** |  |  |  |  |  |  |  |  |
| *Man* | 0.85 | 0.17 | 0.86 | 0.19 | 0.72 | 0.21 | 0.87 | 0.13 |
| *Woman* | 0.79 | 0.19 | 0.83 | 0.17 | 0.76 | 0.24 | 0.80 | 0.19 |
| **Marital status** |  |  |  |  |  |  |  |  |
| *Married* | 0.80 | 0.18 | 0.83 | 0.17 | 0.76 | 0.25 | 0.82 | 0.18 |
| *Single* | 0.79 | 0.20 | 0.84 | 0.18 | 0.75 | 0.19 | 0.79 | 0.20 |
| **Health education** |  |  |  |  |  |  |  |  |
| *Assistant nurse* | 0.80 | 0.18 | 0.83 | 0.17 | 0.75 | 0.24 | 0.82 | 0.17 |
| *Other education* | 0.80 | 0.19 | 0.83 | 0.16 | 0.75 | 0.24 | 0.80 | 0.22 |
| **Tenure and age** |  |  |  |  |  |  |  |  |
| *Up to five years of experience* | 0.83 | 0.18 | 0.85 | 0.16 | 0.71 | 0.26 | 0.83 | 0.20 |
| *More than five years of experience & ≤ 35 years of age* | 0.83 | 0.17 | 0.83 | 0.20 | 0.73 | 0.27 | 0.83 | 0.17 |
| *More than five years of experience & 36-54 years of age* | 0.76 | 0.20 | 0.82 | 0.17 | 0.76 | 0.25 | 0.78 | 0.20 |
| *More than five years of experience & ≥ 55 years of age* | 0.76 | 0.18 | 0.81 | 0.18 | 0.81 | 0.15 | 0.82 | 0.17 |
| **Control at work** |  |  |  |  |  |  |  |  |
| *Low* | 0.79 | 0.18 | 0.81 | 0.18 | 0.76 | 0.23 | 0.79 | 0.21 |
| *High* | 0.82 | 0.17 | 0.85 | 0.16 | 0.74 | 0.25 | 0.84 | 0.17 |
| **Social support at work** |  |  |  |  |  |  |  |  |
| *Low* | 0.77 | 0.20 | 0.81 | 0.18 | 0.73 | 0.24 | 0.78 | 0.22 |
| *High* | 0.82 | 0.16 | 0.84 | 0.17 | 0.78 | 0.23 | 0.83 | 0.17 |

There were 1,076 participants before the pandemic and 563 participants during the pandemic with eligible answers to EuroQol 5 dimensions questions and to the workload questions.
SD= Standard deviation.

**Supplementary Table 4.** Diagnostics of the inverse probability weighting estimates.

|  | **Unweighted^a^** | | | **Weighted^b^** | | |
| --- | --- | --- | --- | --- | --- | --- |
|  | *AbsDiff^c^* | *SDev^d^* | *SDiff^e^* | *AbsDiff^c^* | *SDev^d^* | *SDiff^e^* |
| **Education** | 0.064 | 0.439 | 6.4% | 0.002 | 0.436 | 0.5% |
| **Marital status** | 0.004 | 0.458 | 0.9% | 0.001 | 0.458 | 0.3% |
| **Gender** | 0.041 | 0.356 | 11.6% | 0.001 | 0.365 | 0.2% |
| **Age** | 1.136 | 13.2 | 8.6% | 1.128 | 13.2 | 8.5% |
| **Tenure** |  |  |  |  |  |  |
| *At most one year* | 0.044 | 0.231 | 19.2% | 0.044 | 0.231 | 19.0% |
| *1*–*5 years* | 0.025 | 0.449 | 5.4% | 0.045 | 0.452 | 10.0% |
| *6-*–*15 years* | 0.025 | 0.464 | 5.4% | 0.020 | 0.464 | 4.3% |
| *More than 15 years* | 0.005 | 0.476 | 1.1% | 0.022 | 0.475 | 4.6% |
| **Tenure and age** |  |  |  |  |  |  |
| *Up to five years of experience* | 0.020 | 0.473 | 4.2% | 0.003 | 0.475 | 0.3% |
| *More than five years of experience & ≤ 35 years of age* | 0.004 | 0.297 | 1.3% | 0.002 | 0.294 | 0.7% |
| *More than five years of experience & 36-54 years of age* | 0.013 | 0.462 | 2.9% | 0.001 | 0.461 | 0.3% |
| *More than five years of experience & ≥ 55 years of age* | 0.003 | 0.436 | 0.6% | 0.002 | 0.437 | 0.4% |

^a^ Proportions and mean values in unweighted samples are available in Table 2.
^b^ Estimates after inverse probability weighting estimates based on the propensity scores have been applied to balance the groups.
^c^ The estimated absolute difference (AbsDiff) between those with a high workload and those with a normal workload for the variable.
^d^ The standard deviation (SDev) pools those with high workload (“treatment”) and those with normal workload (“control”).
^e^ The absolute value of the standardised difference (SDiff) is expressed as a percentage.

**Supplementary Table 5.** Effect of high workload on health-related quality of life, as measured with risk difference, depending on workload definition (n = 548)

|  | | **Workload index** | | | |
| --- | --- | --- | --- | --- | --- |
| **Health measure** | | **Median>=3^a^ (n=171)** | **Mean >3^a^ (n=48)** | | **Mean >2.5 (n=173)** |
| *Quality-adjusted life years^b^* | −0.062* | | | -0.121* | -0.065* |
| *EQ-5D^c^ – Mobility^d^* | 0.048 | | | 0.065 | 0.053* |
| *EQ-5D^c^ - Usual activities^d^* | 0.055* | | | 0.075 | 0.071* |
| *EQ-5D^c^ - Pain/discomfort^d^* | 0.058 | | | 0.206* | 0.078 |
| *EQ-5D^c^ - Anxiety/depression^d^* | 0.210* | | | 0.348* | 0.224* |
| *EuroQol Visual Analogue Scale (EQ-VAS)^b^* | −6.94* | | | -9.72* | -7.82* |

^a^ Absolute risk differences were derived based on propensity scores that represented the probability of having a high workload with gender, tenure, health education, and marital status as covariates.
^b^ A risk difference above 0 means fewer problems with health-related quality of life for those with a high workload than those with a normal workload. n refers to the number of individuals defined with a high workload from the definition

^c^ EuroQol 5 dimensions. Responses are dichotomized to no problems and at least moderate problems. Problems with each of the dimensions were: 43 for mobility, 48 for usual activities, 334 for pain/discomfort and 206 for anxiety/depression.

^d^ A risk difference above 0 means more problem with health-related quality of life for those with a high workload than those with a normal workload.
* Statistical significance at 5% level using Bootstrap technique for derivation of p-value. There were 551 valid responses to EQ-VAS estimates.

**Supplementary Table 6.** Sensitivity analyses based on working hours for participants (n = 548)

|  | | **Working hours** | | | | |
| --- | --- | --- | --- | --- | --- | --- |
| **Health measure** | | **All included (n=548)** | **More than 35 hours a week (n=409)** | | | **More than 30 hours a week (n=402)** |
| *Quality-adjusted life years^b^* | −0.062* | | | -0.067* | -0.072* | |
| *EQ-5D^c^ – Mobility^d^* | 0.048 | | | 0.031 | 0.022 | |
| *EQ-5D^c^ - Usual activities^d^* | 0.055 | | | 0.009 | 0.037 | |
| *EQ-5D^c^ - Pain/discomfort^d^* | 0.058 | | | 0.027 | 0.038 | |
| *EQ-5D^c^ - Anxiety/depression^d^* | 0.210* | | | 0.255* | 0.256* | |
| *EuroQol Visual Analogue Scale (EQ-VAS)^b^* | −6.94* | | | -6.61* | -7.34* | |

^a^ Absolute risk differences were derived based on propensity scores that represented the probability of having a high workload with gender, tenure, health education, and marital status as covariates.
^b^ A risk difference above 0 means fewer problems with health-related quality of life for those with a high workload than those with a normal workload. n refers to the number of individuals defined with a high workload from the definition

^c^ EuroQol 5 dimensions. Responses are dichotomized to no problems and at least moderate problems. Problems with each of the dimensions were for the full sample: 43 for mobility, 48 for usual activities, 334 for pain/discomfort and 206 for anxiety/depression.

^d^ A risk difference above 0 means more problem with health-related quality of life for those with a high workload than those with a normal workload.
* Statistical significance at 5% level using Bootstrap technique for derivation of p-value. There were 551 valid responses to EQ-VAS estimates.
