# Supplementary figures and images for: High workload and its connection to health-related quality of life among in-home care workers in northern Sweden during the Covid-19 pandemic
Source: BMC Public Health. 2026 May 4;26:1468. doi: 10.1186/s12889-026-27512-z (PMC13141269; doi:10.1186/s12889-026-27512-z)

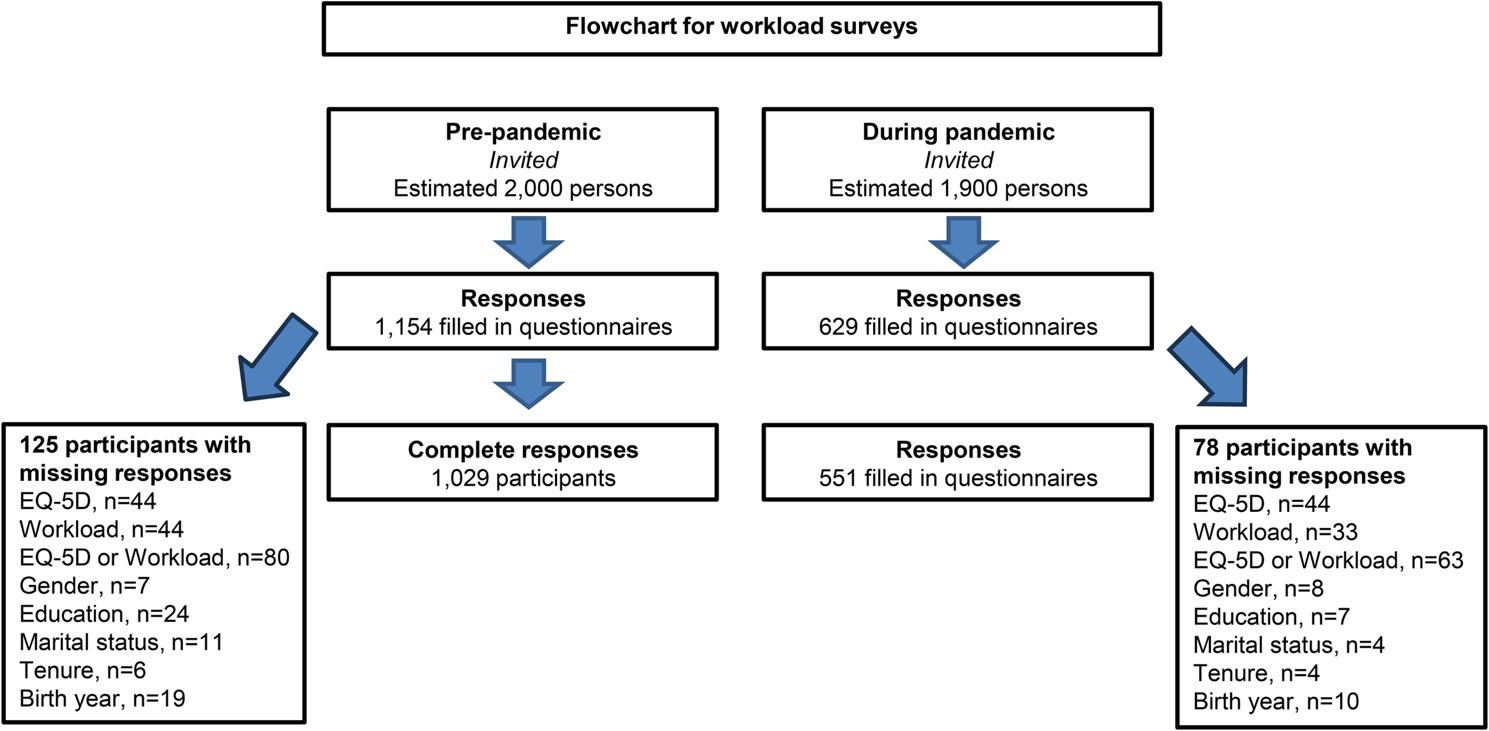


Supplementary Figure 1 Flowchart for participants

Supplement: Supplementary file 3 — Supplementary Material 3. [file 12889_2026_27512_MOESM3_ESM.docx]
